# Monkeypox and dermatological lesions^⋆^

**DOI:** 10.1016/j.abd.2023.04.001

**Published:** 2023-05-22

**Authors:** Amnuay Kleebayoon, Viroj Wiwanitkit

**Affiliations:** a Private Academic Consultant, Samraong, Cambodia; b Research Center, Chandigarh University, Punjab, India

Dear Editor,

We would like to comment on the publication “2022 Mpox (Monkeypox) outbreak: a concise review focused on new features of dermatological lesions”.^1^ Pinto-Pulido et al. Pinto-Pulido et al. pointed out that in addition to epidemiological alterations, clinical variations from the clinical picture previously described in endemic areas have also been recorded. It is unclear if these modifications are brought on by genetic changes in the virus, a unique method of transmission, unique baseline traits of affected patients in comparison to those of endemic nations, or, more likely, a combination of all these elements. Because pseudopustules with a necrotic-hemorrhagic center are uncommon in other dermatological illnesses, Pinto-Pulido et al. noted that a thorough dermatological description of lesions is necessary for a correct diagnosis. We all agree that the key to a successful early diagnosis of monkeypox is a thorough dermatological examination. However, it is challenging to diagnose if there is an atypical appearance, no fever, or no skin lesion at the initial presentation.^2^ Atypical clinical skin lesions can occasionally occur and can be challenging to distinguish from other frequent dermatological issues.^2^ Moreover, there are still issues with both false positive and negative results in the laboratory diagnosis of monkeypox using PCR.^3^ In questionable cases, a thorough clinical history taking, reexamination, and reanalysis of the clinical samples are required.

## Financial support

None declared.

## Authors' contributions

Amnuay Kleebayoon: Ideas, writing, analyzing, approval for submission.

Viroj Wiwanitkit: Ideas, supervision, approval for submission.

## Conflicts of interest

None declared.
